# Active Inhibitor-1 Maintains Protein Hyper-Phosphorylation in Aging Hearts and Halts Remodeling in Failing Hearts

**DOI:** 10.1371/journal.pone.0080717

**Published:** 2013-12-02

**Authors:** Tracy J. Pritchard, Yoshiaki Kawase, Kobra Haghighi, Ahmad Anjak, Wenfeng Cai, Min Jiang, Persoulla Nicolaou, George Pylar, Ioannis Karakikes, Kleopatra Rapti, Jack Rubinstein, Roger J. Hajjar, Evangelia G. Kranias

**Affiliations:** 1 Department of Pharmacology and Cell Biophysics, University of Cincinnati College of Medicine, Cincinnati, Ohio, United States of America; 2 Department of Internal Medicine, Division of Cardiovascular Diseases, University of Cincinnati, Cincinnati, Ohio, United States of America; 3 Cardiovascular Research Center, Ichan School of Medicine at Mount Sinai, New York, New York, United States of America; San Diego State University, United States of America

## Abstract

Impaired sarcoplasmic reticulum calcium cycling and depressed contractility are key characteristics in heart failure. Defects in sarcoplasmic reticulum function are characterized by decreased SERCA2a Ca-transport that is partially attributable to dephosphorylation of its regulator phospholamban by increased protein phosphatase 1 activity. Inhibition of protein phosphatase 1 through activation of its endogenous inhibitor-1 has been shown to enhance cardiac Ca-handling and contractility as well as protect from pathological stress remodeling in young mice. In this study, we assessed the long-term effects of inducible expression of constitutively active inhibitor-1 in the adult heart and followed function and remodeling through the aging process, up to 20 months. Mice with inhibitor-1 had normal survival and similar function to WTs. There was no overt remodeling as evidenced by measures of left ventricular end-systolic and diastolic diameters and posterior wall dimensions, heart weight to tibia length ratio, and histology. Higher phosphorylation of phospholamban at both Ser16 and Thr17 was maintained in aged hearts with active inhibitor-1, potentially offsetting the effects of elevated Ser2815-phosphorylation in ryanodine receptor, as there were no increases in arrhythmias under stress conditions in 20-month old mice. Furthermore, long-term expression of active inhibitor-1 via recombinant adeno-associated virus type 9 gene transfer in rats with pressure-overload induced heart failure improved function and prevented remodeling, associated with increased phosphorylation of phospholamban at Ser16 and Thr17. Thus, chronic inhibition of protein phosphatase 1, through increases in active inhibitor-1, does not accelerate age-related cardiomyopathy and gene transfer of this molecule *in vivo* improves function and halts remodeling in the long term.

## Introduction

Heart failure is the leading cause of death in the United States with more than 550,000 new diagnoses per year [1]. One of the hallmarks of failing hearts is impaired calcium cycling. A key player in cardiac calcium homeostasis is the sarcoplasmic reticulum calcium ATPase (SERCA), which is negatively regulated by a small phosphoprotein, phospholamban [2]. When phospholamban is phosphorylated through either activation of protein kinase A (PKA) or calcium-calmodulin kinase II (CaMKII), the inhibition of the SERCA affinity for calcium by phospholamban is relieved [2]–[4]. Moreover, de-phosphorylation of phospholamban is regulated by protein phosphatase-1 (PP1), which is itself modulated by a regulatory protein called inhibitor-1 (I-1) [5]–[7]. In failing hearts, SERCA activity is reduced [8]–[10], which may be partially attributed to increased inhibition by dephosphorylated phospholamban [4], [11]–[13]. Restoring SERCA levels or decreasing PLN inhibition has been shown to improve function, remodeling and survival [4]. An emerging pathway in modulating PLN activity is inhibition of protein phosphatase 1 (PP1) that is significantly elevated in heart failure. The increases in PP1 activity are partially attributable to a reduction in the levels and activity of its endogenous I-1 [14]–[17]. Thus, the phospholamban associated inhibitor-1/PP1 regulatory complex plays a key role in the compromised or dys-regulated Ca-cycling in failing hearts.

Inhibitor-1 is a 171 amino acid protein containing three phosphorylation sites. Residue threonine-35 is phosphorylated by PKA and enhances the ability of inhibitor-1 to depress PP1 activity [18], [19]. On the other hand, protein kinase C phosphorylates inhibitor-1 at serine-67 and threonine-75, which reduce its inhibitory effects on PP1 [20]–[22]. In human heart failure, the phosphorylation at position 35 and consequently the activity of inhibitor-1 is decreased [15], [17]. To elucidate the functional significance of inhibitor-1 in vivo, we have generated transgenic mouse models with either chronic or inducible expression of constitutively phosphorylated (T35D) and truncated (contains amino acids 1–65) inhibitor-1 (I-1c) in the heart [23], [24]. We observed that conventional or inducible expression of I-1c increased phosphorylation of PLN, enhanced contractility and was cardioprotective to pressure overload, chronic β-adrenergic stimulation, and ischemia-reperfusion injury [23]–[25]. Furthermore, gene transfer of I-1c in rat failing hearts with adenovirus or porcine failing hearts with adeno-associated virus preserved cardiac function and reduced remodeling or scar size [23], [26]. However, there has been a conflicting report indicating that although I-1c improved contractile function in young mice it induced cardiomyopathy upon aging and promoted lethal catecholamine-associated ventricular tachycardia [27]. In that study, I-1c was expressed in the absence of endogenous inhibitor-1, which partially limits the physiological interpretation of the observed findings.

Thus, the present study was designed to evaluate the long-term effects of increased I-1c expression in the wild-type background (in the presence of endogenous I-1) under the physiological stress of aging. Expression of I-1c was induced in the adult mouse heart and assessment of cardiac remodeling, survival and susceptibility to acute stress revealed no differences between I-1c and WT mice up to 20-months of age. Furthermore, gene transfer of I-1c in failing rat hearts improved contractility and halted remodeling in the long-term.

## Methods

### Generation of Mice

Transgenic mice with inducible expression of a pseudophosphorylated and truncated I-1 were generated and genotyped as previously described [24]. Mice were fed doxycycline (TestDiet, Richmond, IL) for four weeks after birth at which time the mice were switched to a standard chow diet (TestDiet). Male and female mice were used in this study and monitored up to 20 months of age. This study was carried out in strict accordance with the recommendations in the Guide for the Care and Use of Laboratory Animals of the National Institutes of Health [28], [29]. The protocols were approved by the Committees on Animal Care and Use of the University of Cincinnati (A3295-01) and Mount Sinai (LA10-00227). All surgery was performed under ketamine/xylazine anesthesia, and all efforts were made to minimize suffering.

### Gravimetric Analysis

Mice were anesthetized using sodium pentobarbitol (60 mg/kg; Ovation Pharmaceuticals, Inc., Deerfield, IL), and the hearts and lungs were removed from the chest cavity. The tissues were rinsed in Dulbecco's phosphate buffered saline, blotted with filter paper, and then weighed. Hearts were snap frozen in liquid nitrogen for immunoblotting experiments. The skeletal muscle of the lower hind limbs was dissected out, and the length of the left tibia was measured.

### Histology

Twenty-month aged mice were anesthetized and the hearts extracted as described above. The hearts were fixed in 10% formalin (Sigma, St. Louis, MO) for 48 hours. The heart was cut into anterior and posterior halves and placed into tissue cassettes. Tissue embedding into paraffin, sectioning (5 µm thick), hemotoxylin and eosin staining, and Masson's trichrome staining were all performed by the Pathology Research Core at Cincinnati Children's Hospital Medical Center.

### Wheat Germ Agglutinin Assay

To assess cardiomyocyte cross-sectional area in WT and I-1c hearts, paraffin-embed sections (5 µm) were deparaffinized by sequentially immersing in xylene, dehydrated in 100% ethanol, 95% ethanol, 80% ethanol, 70% ethanol and washed with distilled water. Then, antigen retrieval was performed in 1 mM sodium citrate buffer at 90°C for 6 minutes. After being cooled to room temperature and washed three times with PBS, sections were incubated with Alexa Fluro488 conjugated-Wheat Germ Agglutinin (5 µg/mL; Invitrogen) at 37°C for 60 minutes. The sections were then scanned at 400× magnification using a fluorescent microscope (Olympus BX51), and the cardiomyocyte cross-sectional area was analyzed by software Image-Pro Plus5.1.

### Immunoblotting

Hearts were homogenized in cell lysis buffer (Cell Signaling, Danvers, MA), supplemented with 34 µM phenylmethylsulfonyl fluoride (PMSF), protease inhibitor cocktail (Roche Diagnostics Corporation, Indianapolis, IN), and phosphatase inhibitor cocktails I and II (Calbiochem, Billerica MA) using 1.0 mm silica beads (Biospec Products, Inc, Bartlesville, OK) and a Precellys 24 tissue homogenizer (Bertin technologies, France). Homogenates were centrifuged at 12,000 rpm for 10 min at 4°C, and the supernatant was collected for subsequent experiments. Protein samples were separated by SDS-PAGE, and then transferred to nitrocellulose membranes. Membranes were probed with the following antibodies: Ser-16 phospho-phospholamban and Thr-17 phospho-phospholamban (Badrilla, Leeds, UK), Ser-2808 phospho-ryanodine receptor and Ser-2815 phospho-ryanodine receptor (Badrilla), phospholamban (Pierce, Rockford, IL), ryanodine receptor (Affinity Bioreagents, Golden, CO), inhibitor-1 (Covance Inc., Denver, PA), sarcoplasmic reticulum calcium ATPase, and GAPDH (Pierce). Horseradish peroxidase linked anti-mouse or anti-rabbit secondary antibodies (GE Healthcare, Pittsburgh, PA) were used, followed by developing with either enhanced chemiluminescence (GE Healthcare) or SuperSignal (Thermo Scientific, Rockford, IL). Films were scanned and analyzed by densitometry using ImageQuant 5.2 (Molecular Dynamics).

### Quantitative PCR

Total RNA was isolated from infected rat hearts and reverse transcribed as previously described (26). Quantitative real-time PCR was performed using the SYBR green system and forward and reverse primers to AAV9.I-1c as previously published (26).

### Electrocardiography

Mice at 20 months of age were anesthetized using isoflurane. The mice were placed in a supine position and the body temperature maintained using a warming pad. Stainless steel electrodes were placed subcutaneously on the upper right limb and lower left limb. The surface ECG was recorded using the PowerLab system (AdInstruments, Colorado Springs, CO) as previously described [30]. Basal rhythm was recorded for 5 minutes and then mice were injected subcutaneously with 0.3 mg/kg isoproterenol.

### Echocardiography

#### Mice

Animals were anesthetized with direct inhalation of isoflurane (1.5–2%) and continuous ECG tracings were recorded. Images were obtained using a VisualSonics Vevo 2100 imaging system (Toronto, Canada) with an MS400 (30 MHZ centerline frequency) probe. Both parasternal long axis (PSLAX), and short axis (SAX) views in B-mode were obtained. The B-mode guided M-mode view at the papillary muscle level was collected for the evaluation of end-diastolic and end-systolic left ventricular wall and chamber dimensions in the PSLAX view. Pulse wave and tissue Doppler was obtained from the PSLAX view. The E (early diastolic flow, MV E) and A (atrial contraction flow, MV A) waves were measured as well as the tissue Doppler obtained E′ and A′. The E wave, E/A, E/E′ and E′/A′ ratio were used to evaluate diastolic function. Using the PSLAX M-mode, the end systolic and diastolic wall and chamber dimensions were measured and used to calculate the ejection fraction (EF), fractional shortening (FS) and left ventricular volumes. B-mode images from the SAX view were used to calculate radial displacement and longitudinal strain using VevoStrain software (Vevo 2100, v1.1.1 B1455, Visualsonic, Toronto, Canada). The circumferential strain and radial displacement were averaged for assessment of global function by the same investigator who was blinded as to the type of the subjects.

#### Rats

Echocardiography was performed under sedation by intraperitoneal ketamine (40–80 mg/kg). Short-axis parasternal views of the LV at the mid-papillary level and long-axis parasternal views of the LV were obtained using a Vivid 7 machine with a 13 MHz linear array probe (General Electric, New York, NY) at a frame rate ranging from 25 to 28 frames/second for 2D echocardiography. M-mode echocardiography was performed on short-axis parasternal views to obtain LV end-diastolic and end-systolic diameters. The frame rate for M-mode echocardiography was 48 frames per second. In 2D and M-mode LV cavity measurements, care was taken to exclude papillary muscles from the LV wall.

### In Vivo Gene Transfer

**Figure 1:**
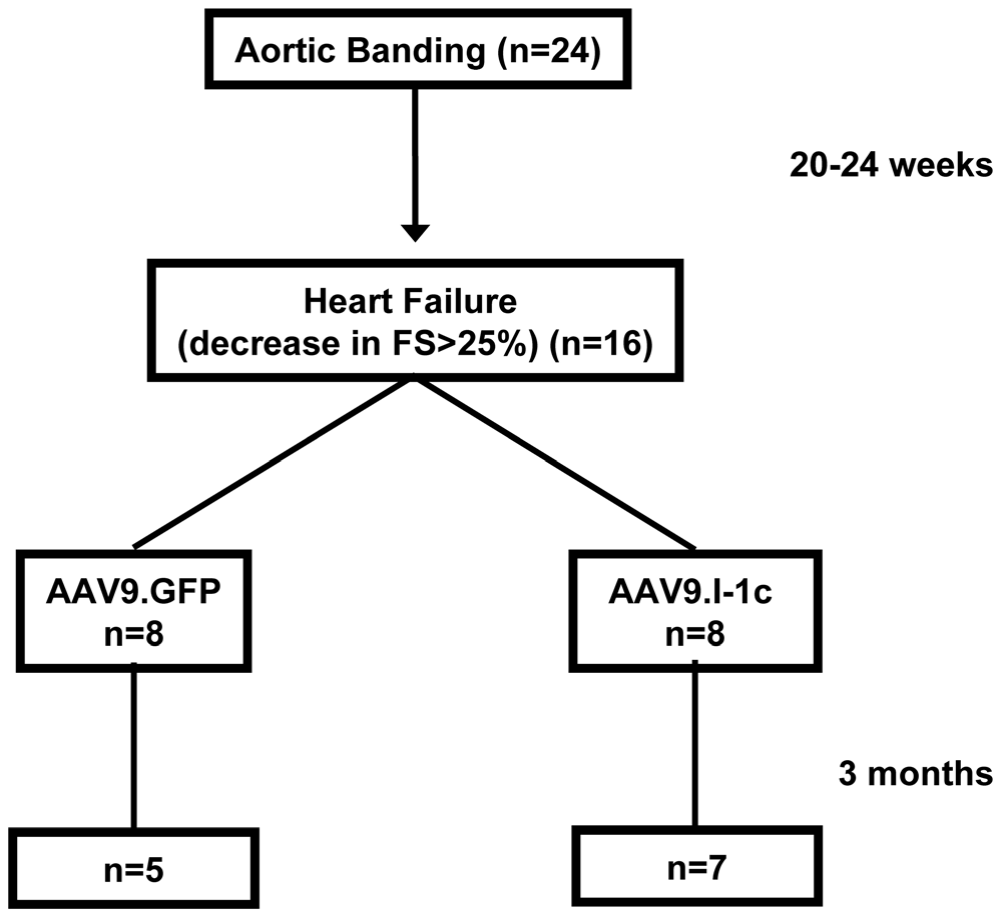
Study design. Twenty-four rats underwent aortic banding. After 20–24 weeks, sixteen rats with FS less than 25% were split into groups of eight receiving AAV9.GFP and AAV9.I1c. Five animals out of eight, receiving AAV9.GFP, survived until euthanization after 90 days, whereas seven out of eight survived in the AAV9.I1c group. Sham procedures were processed in parallel.

AAV9.I-1c and AAV9-GFP were generated as previously described [26]. Four week old Wistar rats (70–80 g) were obtained from Charles River Laboratories (Wilmington, MA) and aortic constriction was performed as previously described [31]. All animals survived the initial operation. Upon a decrease in fractional shortening of over 25% (20–24 weeks post-TAC), the animals were randomized in two groups: one group of 8 rats received I-1c adeno-associated virus (AAV9.I-1c) and another group of 8 rats received control AAV9 with a reporter gene (AAV-GFP) at a concentration of 10^11^ vg (which yields maximal expression in rats within 30 days of injection) through tail injection [32]. Sham operated animals were processed in parallel. Three months following gene transfer, pressure measurements were performed [31], [33]. The final number of animals was: 5 in AAV9.GFP and 7 in AAV.9-I-1c groups, as some died of progressive heart failure (Figure 1). We determined the following parameters: systolic pressure, end-diastolic pressure, peak LV pressure rates of rise (+dP/dt) and fall (−dP/dt) and Tau (time constant of isovolumic relaxation).

### Statistical Analysis

Data are presented as the mean +/− standard error of the mean. For comparisons between two groups, Student's t-test was employed. A P-value less than 0.05 was taken as significant. Survival data was assessed by Chi Square analysis. All statistical analysis was performed using GraphPad Prism 4 software (La Jolla, CA).

## Results

### Aging and Survival of Mice with Inducible Expression of I-1c in the Heart

**Figure 2:**
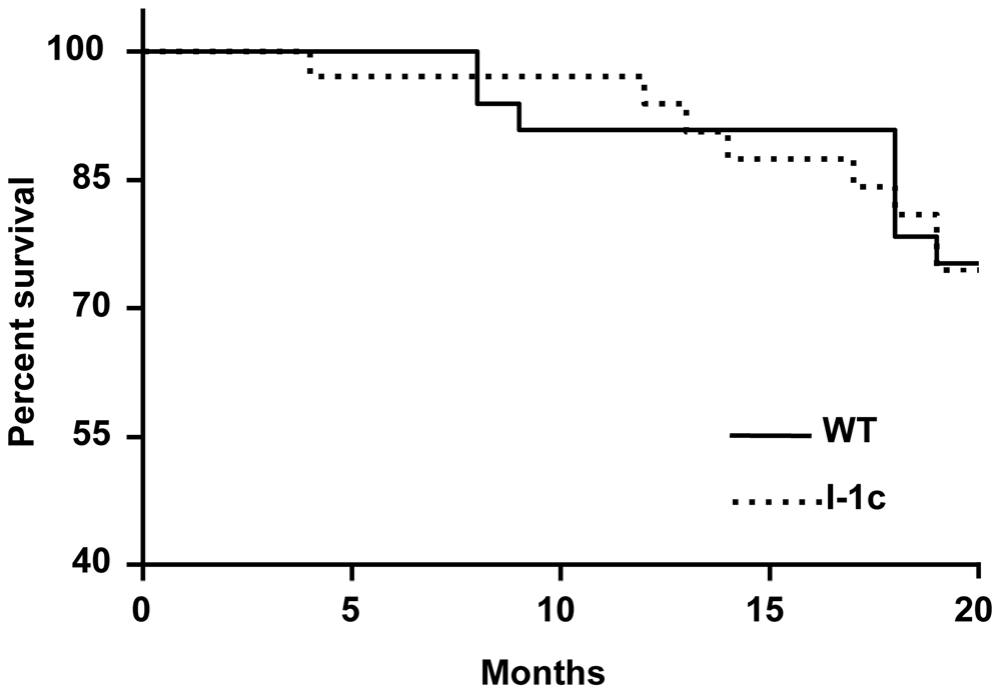
Survival rates in I-1c expressing and WT mice. The percentages of I-1c and WT mice alive at each time point throughout the 20 month aging study were plotted as Kaplan-Meier survival curves. The survival curves for WT and I-1c mice were similar averaging 75% survival at 20 months with a Chi-square value of 0.01. n = 33 mice for WT and 34 mice for I-1c.

Inducible expression of I-1c increased basal cardiac contractility via enhanced phosphorylation of phospholamban and increased calcium uptake by the SR Ca-ATPase (SERCA). These transgenic mice also exhibited cardioprotection from ischemia-reperfusion injury [24]. To determine the long-term effects of I-1c in vivo under the physiological stress of aging, mice with inducible expression of I-1c were evaluated in parallel with isogenic WT mice up to 20 months of age. There was no difference in survival rates between I-1c and WT mice with ∼75% of I-1c and WT mice surviving to 20 months of age (Figure 2). Furthermore, there was no increased incidence of gross age-related symptoms including lethargy, tumors, seizures, or sudden death.

### In Vivo Cardiac Function and Remodeling of I-1c Mice

**Figure 3:**
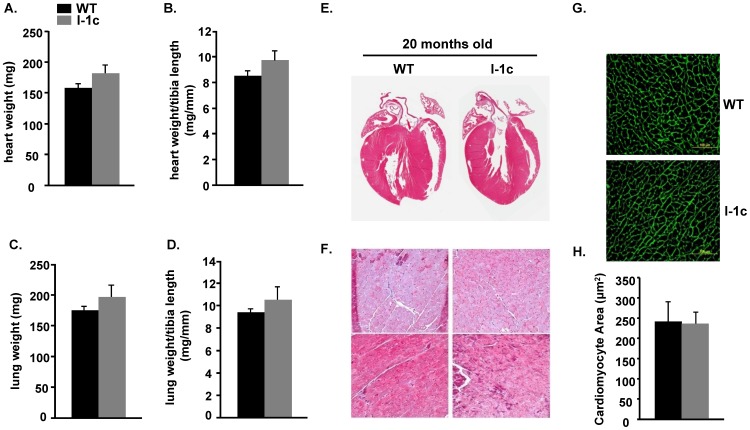
Gravimetric analysis and histological evaluation of tissues from 20 month old I-1c and WT mice. Weights of: A) hearts; B) heart to tibia length; C) lungs; and D) lung to tibia length were assessed and no significant differences were observed. Data represent the mean ± SE. n = 28 for WT and 24 for I-1c mice. Hemotoxylin and eosin staining of cardiac tissue sections from 20 month old WT and I-1c mice revealed no differences in morphology. F) Masson's trichrome staining (200× magnification) showed no significant fibrosis in either WT or I-1c mice. G) Representative myocardial sections show cardiomyocyte cross-sectional area (Alexa Fluor 488-tagged wheat germ agglutinin labeling, 400× magnification). H) Quantitative results indicate similar average cross-sectional area in aging WT and I-1c cardiomyocytes (n>450 myocytes).

In vivo cardiac function and remodeling were followed longitudinally for I-1c and WT mice up to 20 months of age. Previous studies indicated that cardiac contractility was significantly higher in I-1c mice at 3 months of age, assessed by in vivo catheterization or ex vivo Langendorf perfusion [24]. However, echocardiography showed similar ejection fraction and fractional shortening between these groups at a young age (Table 1), suggesting that neurohormonal factors may mask the contractile differences in intact mice. Upon aging to 20 months, the contractile or geometrical parameters, as evidenced by left ventricular end diastolic diameter (LVEDD), left ventricular end systolic diameter (LVESD), left ventricular posterior wall dimensions (LVPW), and circumferential strain remained similar between WT and I-1c mice (Table 1). Diastolic function, mitral valve early diastolic flow (MV E/E′), measured by tissue Doppler was not significantly different between WT and I-1c mice (Table 1). We then used gravimetric analysis and hematoxylin and eosin staining of fixed cardiac tissue sections to further evaluate cardiac remodeling in 20 month old WT and I-1c hearts. There were no differences in heart weight or heart weight to tibia length ratios between I-1 and WTs (Figure 3A and B). Furthermore, there were no signs of heart failure or lung congestion in these mice. The lung weight or lung weight to tibia length ratios were similar (Figure 3C and D). Additionally, hematoxylin and eosin staining did not reveal any gross morphological changes between WT and I-1c hearts (Figure 3E) and assessment of myocyte cross-sectional area confirmed these results (Figure 3G and H). Moreover, Masson's trichrome stained cardiac sections showed no appreciable fibrosis in either WT or I-1c at 20 months (Figure 3F).

**Table 1:** Echocardiographic Parameters.

	3 months		20 months	
	WT (n = 18)	I-1c (n = 17)	WT (n = 15)	I-1c (n = 16)
EF (%)	62.58±2.79	58.67±1.43	63.88±2.63	62.22±2.75
FS (%)	33.78±2.07	30.24±0.97	34.47±1.79	33.46±2.09
LVESD (mm)	2.37±0.14	2.69±0.09	2.40±0.14	2.38±0.09
LVEDD (mm)	3.55±0.14	3.87±0.10	3.65±0.15	3.59±0.09
LVPWS (mm)	1.50±0.08	1.54±0.13	1.60±0.08	1.61±0.08
LVPWD (mm)	1.22±0.08	1.24±0.11	1.27±0.09	1.22±0.07
MV E/É	−44.73±4.08	−39.99±15.72	−39.93±4.17	−37.69±2.56
Circumferential Strain	−20.21±1.03	−21.55±0.84	−21.84±1.46	−22.61±1.36
Radial Displacement	0.40±0.02	0.45±0.02	0.44±0.03	0.45±0.03

Echocardiographic parameters obtained in young (3 mos) and aging (20 mos) WT and I-1c mice. Data represent the mean ± SE. Ejection fraction (EF), fractional shortening (FS), left ventricular end systolic diameter (LVESD), left ventricular end diastolic diameter (LVEDD), systolic left ventricular posterior wall thickness (LVPWS), and diastolic left ventricular posterior wall thickness (LVPWD), and ratio of mitral valve early diastolic flow via tissue Doppler (MV E/E′).

### Phosphorylation of Sarcoplasmic Reticulum Protein Substrates in Aged Hearts

**Figure 4:**
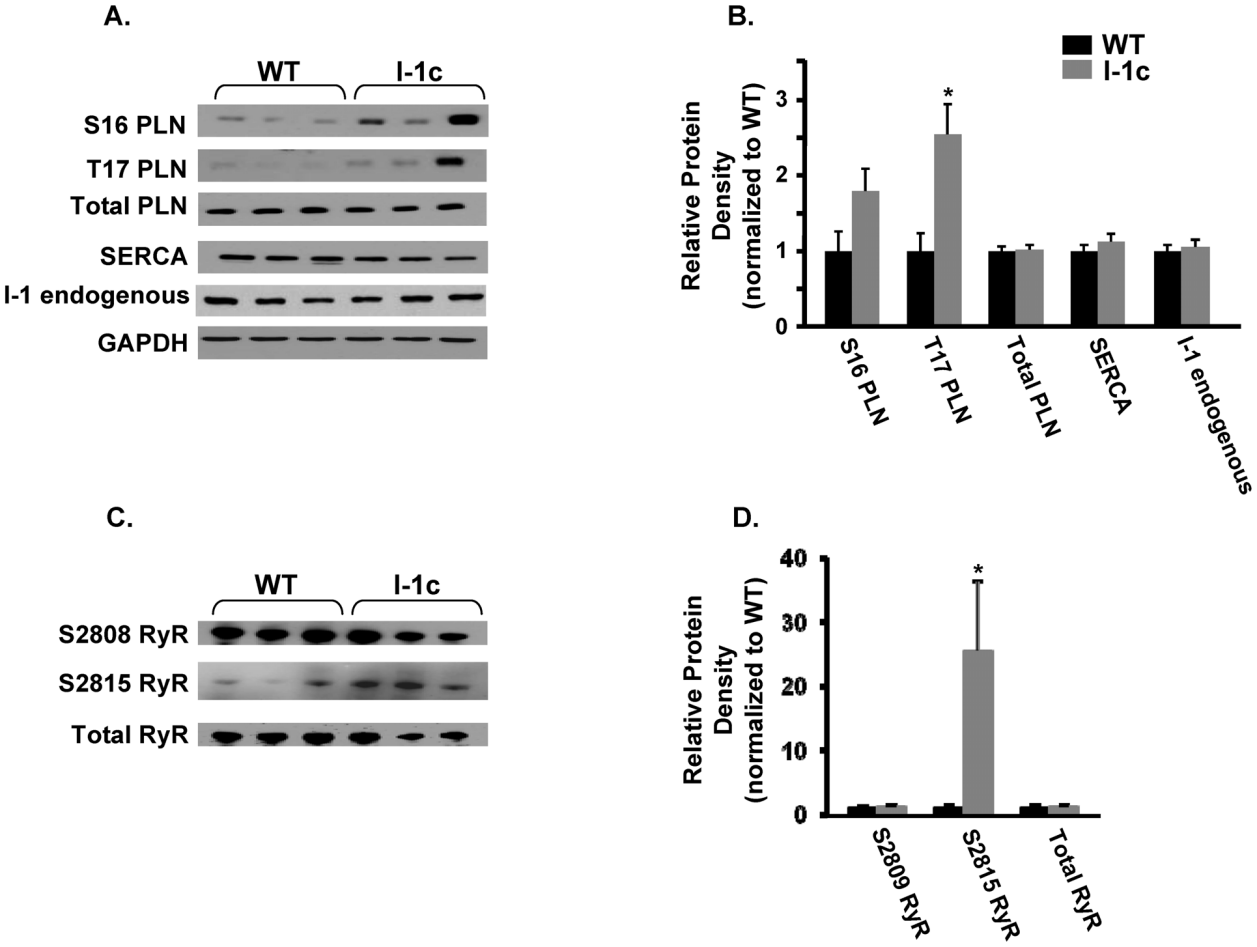
Ca handling proteins and phosphoproteins in 20 month old I-1c and WT hearts. A and C) Representative blots showing the expression of Ser-16 phospho-phospholamban (S16 PLN), Thr-17 phospho-phospholamban (T17 PLN), phospholamban (total PLN), sarcoplasmic reticulum Ca ATPase (SERCA), endogenous (full-length) inhibitor-1, Ser-2808 phospho-ryanodine receptor (S2808 RyR), Ser 2815 phospho-ryanodine receptor (S2815 RyR), and ryanodine receptor (RyR). B and D) Quantitative analysis of protein expression in I-1c hearts relative to WTs. Protein levels were normalized to the loading control glyceraldehyde 3-phosphate dehydrogenase (GAPDH). Bars represent the mean ± SE. n = 6 hearts. * indicates P-value <0.05.

Overexpression of I-1c has been previously shown to increase phosphorylation of phospholamban in young hearts [23], [24], [27]. Therefore, we measured the phosphorylation levels of several proteins central to calcium handling and contractility at 20 months of age. We found that phosphorylation of phospholamban at both Ser-16 and Thr-17 sites was elevated in I-1c hearts, while total phospholamban, SERCA and endogenous inhibitor-1 expression was unchanged (Figures 4A and B). Interestingly, the increases in phospholamban phosphorylation were similar to those in young I-1c hearts [24], confirming the sustained overexpression of I-1c through the aging process. Moreover, the ryanodine receptor had increased phosphorylation at its calmodulin kinase II site (Ser-2815) but not at the PKA site (Ser-2808) in I-1c hearts compared to WTs (Figures 4C and D). Assessment of the endogenous I-1 levels indicated no alterations upon aging to 20 months (data not shown).

### Electrocardiography of I-1c Mice Under β-Adrenergic Stress

**Figure 5:**
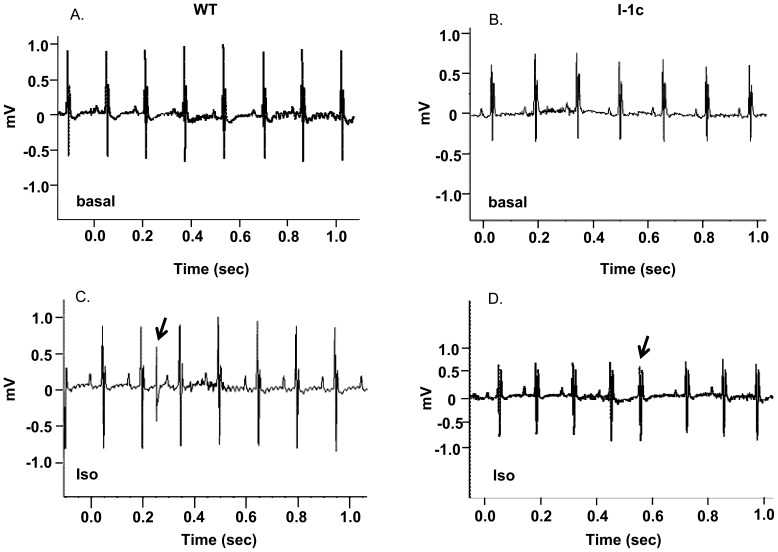
Electrocardiography in aged WT and I-1c mice under isoproterenol stress. Twenty month old WT and I-1c mice were anesthetized with isoflurane and ECG recorded. Representative recordings (1 second (sec)) show that both WT and I-1c exhibited normal sinus rhythm without ectopy under baseline conditions (A and B) and in response to subcutaneous injection of isoproterenol (0.3 mg/kg) premature ventricular contractions (indicated by arrow) were noted in both groups (C and D).

Since the I-1c model had increased phosphorylation of ryanodine receptor at the CaMKII site, which has been shown to be associated with arrhythmias [27], we subjected the 20-month old mice to acute stress and examined their susceptibility to arrhythmogenesis. Surface ECG recordings were obtained under basal conditions and after acute catecholaminergic stimulation with isoproterenol (0.3 mg/kg). Under basal conditions, no sustained arrhythmias were observed in WT mice, and only 1 out of 6 I-1c mice showed abnormal cardiac rhythm in the form of premature ventricular contractions. After injection of isoproterenol, 2 out of 10 WT mice showed sustained arrhythmias (one exhibited recurring premature ventricular contractions and the other sinus bradycardia) and 1 out of 6 I-1c mice showed premature ventricular contractions (Figure 5). Thus, aging mice with increased I-1c expression were not susceptible to arrhythmias upon β-adrenergic stress.

### I-1c Delivery to the Myocardium by the Cardiotropic AAV9 Vector

Since studies in our transgenic model suggested that I-1c does not elicit adverse effects under the physiological stress of aging, we then investigated whether long-term expression of this molecule could improve hemodynamics in the setting of pre-existing heart failure. Thus, we utilized a rat model of pressure overload, which exhibits characteristics of heart failure by 20–24 weeks post-banding [31]. When decreases of more than 25% in left ventricular fractional shortening were observed, gene transfer was performed (Figure 1). Adeno-associated virus type 9 (AAV9) has been shown to have cardiac tropism in rodents with high level of ventricular expression, when injected through the tail vein [32], [34], [35]. Cardiac function was studied invasively prior to sacrificing the infected animals at 3 months post-infection. Remarkably, gene transfer of the active inhibitor-1 significantly improved the rate of pressure rise (+dP/dt), while left ventricular function was decreased in the failing control group (Table 2) despite the presence of the aortic band. Diastolic parameters were also normalized, as evidenced by restoration of the maximal rate of decline of left ventricular systolic pressure (−dP/dt) and the time course for pressure decline, measured by tau, the isovolumic relaxation constant (Table 2).

**Table 2:** Hemodynamic Parameters.

Treatment	HR (min-1)	LVSP (mmHg)	LVEDP (mmHg)	+dP/dt	-dP/dt	Tau (msec)
Control Sham (n = 10)	410±24	98±4	6±2	8,772±83	−6,032±34	14±3
Failing + AAV9.GFP (n = 5)	392±43	122±19	22±6	4,078±60	−3,154±56	24±5
Failing + AAV9.I-1c (n = 7)	401±38	134±24	16±5	6,284±73*	−5,294±49*	19±6

Hemodynamic Parameters obtained from rats treated with AAV9.I-1c at 3 months after aortic banding: heart rate (HR), left ventricular systolic pressure (LVSP), left ventricular end-diastolic pressure (LVEDP), the peak LV pressure rate of rise (+dP/dt), the maximal rate of decline of left ventricular systolic pressure (−dP/dt), time constant of isovolumic relaxation (tau). *p<0.05 compared to AAV9.GFP.

**Figure 6:**
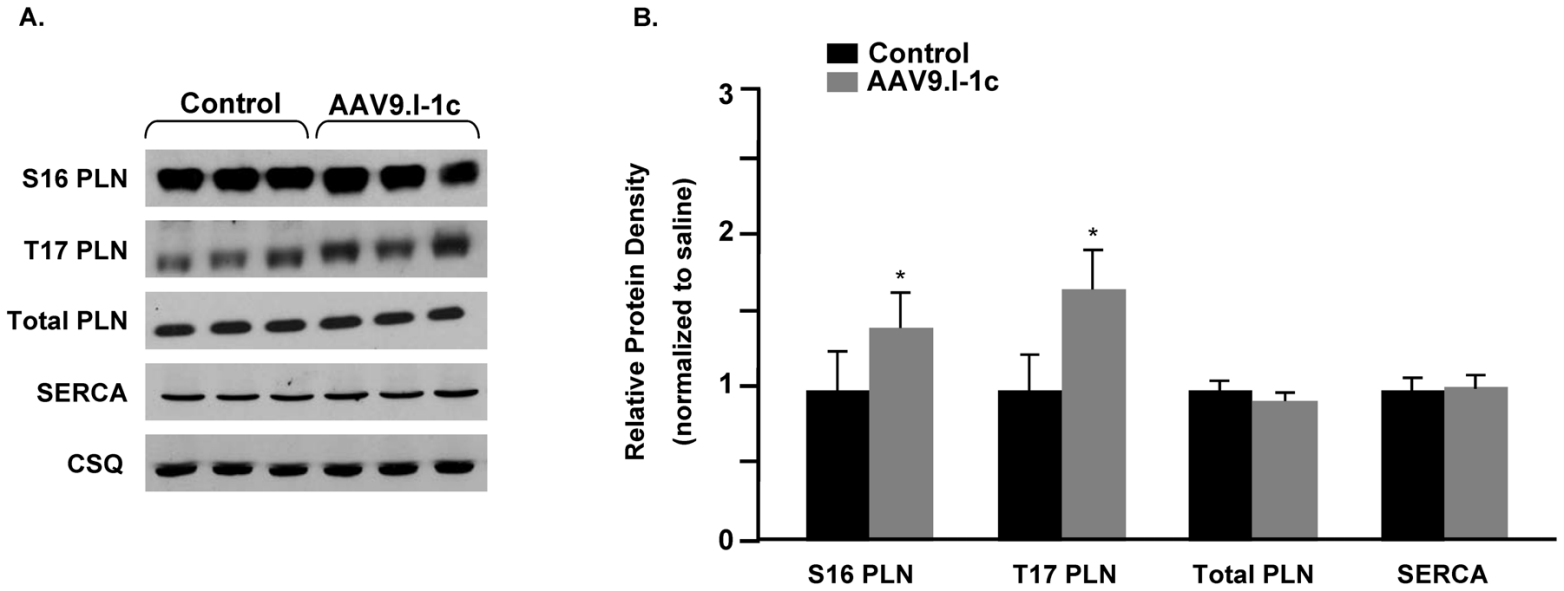
Phospholamban phosphorylation in AAV9.I-1c treated rat hearts. A) Representative immunoblots showing the protein expression of Ser-16 phospho-phospholamban (S16 PLN), Thr-17 phospho-phospholamban (T17 PLN), phospholamban (total PLN), and sarcoplasmic reticulum Ca ATPase (SERCA) in AAV9.I-1c treated rat hearts at 3 months post-injection. B) Quantitative analysis of protein expression relative to control rat hearts. Protein levels were normalized to the loading control calsequestrin (CSQ). Bars represent the mean ± SE. n = 3 hearts. * indicates P-value<0.05.

At the end of the study period, fractional shortening (FS) as determined by echocardiography was significantly higher in I-1c treated animals (49.2±3.3%) than in failing animals (33.8±6.2%, P<0.05, Table 3), consistent with improved contractility. Expression of I-1c, assessed by transcript levels, was 4.0×10^4^ copies/100 ng RNA and phosphorylation of phospholamban was significantly enhanced at both S16 and T17 sites in I-1c treated rats, while sarcoplasmic reticulum Ca ATPase and total phospholamban levels were not changed (Figure 6A and B). There were no alterations in the phospho- and total levels of ryanodine receptor either (data not shown). Furthermore, cardiac remodelling associated with increases in LVDD and LVSD was significantly halted in I-1c treated animals and these parameters were similar to those in control animals. In summary, the presented data suggest that gene therapy with I-1c may induce functional improvements and halt remodelling in failing rat hearts.

**Table 3:** Echocardiographic Parameters.

Treatment	AW (mm)	PW (mm)	LVDD (mm)	LVSD (mm)	FS (%)
Control Sham (n = 10)	1.83±0.11	1.86±0.13	6.7±0.33	3.2±0.22	52.2±4.1
Failing + AAV9.GFP (n = 5)	2.66±0.11	2.68±0.12	9.1±0.40	6.3±0.42	33.8±6.2
Failing + AAV9.I-1c (n = 7)	2.24±0.15	2.18±0.09*	7.2±0.34*	3.6±0.33*	49.2±3.3*

Echocardiographic parameter measurements of rats treated with AAV9.I-1c at 3 months after aortic banding: Anterior wall thickness (AW), Posterior wall thickness (PW), left ventricular diastolic dimension (LVDD), left ventricular systolic dimension, fractional shortening (FS). *p<0.05 compared AAV9.GFP.

## Discussion

This study presents evidence that chronic increases of constitutively active inhibitor-1 (I-1c) to suppress cardiac PP1 activity and enhance PLN phosphorylation are well-tolerated through the aging process without compromising survival, increasing remodeling or eliciting arrhythmias under stress conditions. Previous studies have shown that overexpression of I-1c enhances cardiac Ca-cycling and contractility in young mouse hearts [23], [24], [27] and maintains function as well as geometry under pathological conditions associated with pressure-overload, ischemia/reperfusion injury, and prolonged β-adrenergic stimulation [23]–[25]. The current findings indicate that sustained increases of I-1c activity by inducing its expression in the adult heart, do not elicit any adverse effects during the aging process. Assessment of cardiac function and geometric parameters at 20-months of age, indicated that these parameters were similar between WT and I-1c mice.

A previous report used a model with inducible I-1c expression in the null background and found that although contractility was increased in young mice, the animals developed a spontaneous cardiomyopathic phenotype upon aging [27]. The reason for this apparent discrepancy may be attributable to several factors. First, the previous model had much higher I-1c expression levels compared to ours (25X vs. 1.65X) [24], [27]. Thus, it is possible that high I-1c levels saturated the SR associated PP1 and localized to other targets, contributing to cardiac dysfunction. In addition, the absence of the endogenous gene and the high levels of I-1c expression may elicit compensatory responses promoting cardiomyopathy upon aging. Secondly, our I-1c model is on the FVB/N background whereas the previous model is in the C57L/B6 background [27]. It has been well recognized that genetic background or mouse strain can significantly impact cardiac contractility and Ca handling [36]–[39]. In fact, FVB/N mice have been reported to exhibit increased left ventricular ejection fraction and fractional shortening with significantly faster heart rate and lack of diurnal variation in heart rate, compared with C57L/B6, BALB/C and SV129 strains [36]. Additionally, the C57L/B6 mouse is more susceptible to remodeling under cardiac stresses. C57L/B6 mice have an earlier onset and more pronounced impairment in contractile function, with corresponding left and right ventricular dilatation, fibrosis, change in expression of hypertrophy markers upon aortic banding [37], which is a model of left ventricular hypertrophy associated with increased hemodynamic load and sustained β-adrenergic stimulation. In contrast, FVB/N mice are relatively resistant and show delayed transition to decompensated heart failure [39]. Furthermore, a study by Berthonneche *et al*., 2009 [38] on cardiovascular-related phenotypes upon β-adrenergic challenge in 23 strains of mice with different genetic backgrounds, indicated compartmental and strain-specific cardiac sensitivity to isoproterenol, with atria responding at lower concentrations than ventricles in the majority of the strains. Although the biological mechanisms underlying differential sensitivities to chronic β-stimulation are not known, the authors speculated that it might reflect distinct and strain-specific distributions of atrial and ventricular β-adrenergic receptors and/or differential downstream signaling pathways. These findings further indicate that responses to β-adrenergic stimulation are determined by complex genetic blueprints and modifiers in different genetic backgrounds [38]. Thus, we speculate that the function of I-1, an amplifier of the β-adrenergic pathway, would be similarly impacted by genetic background.

Furthermore, we observed that phosphorylation of phospholamban at both ser-16 and thr-17 and phosphorylation of RyR at ser-2815 was higher in aging I-1c mice, compared to WTs. Increased CAMK-phosphorylation of RyR at ser-2815 was also observed in the previous model with high I-1c expression and this was suggested to contribute in cardiac arrhythmias [27]. However, we did not obtain susceptibility to catecholaminergic stress in our 20-month old I-1c mice even in the phase of increased phosphorylation of Ser-2815 in RyR. The reason for this apparent discrepancy may be related to differences in the levels of I-1c expression, the absence or presence of endogenous I-1, strain-specific distributions of β-adrenergic receptors and/or differential downstream signaling pathways, as outlined above. In addition, strain differences in Ca handling have been reported. In fact, it has been shown that C57/BL6 mice have increased Ca spark frequency and SR Ca leak compared to FVB/n mice [36]. Thus, when evaluating the effects of gene alterations/activity in the heart, genetic background may play an essential role in the observed phenotypes.

Importantly, AAV9 gene-transfer of I-1c in rats with heart failure preserved contractile performance and mitigated the adverse remodeling post-aortic constriction in the long-term. Invasive hemodynamics indicated that the rates of contraction and relaxation as well as the time constant for relaxation, tau, were significantly improved in the I-1c treatment group. Accordingly, fractional shortening assessed by echocardiography in the I-1c group exhibited restored values to those observed in the sham group. Furthermore, cardiac remodeling was halted as revealed by the left ventricular systolic and diastolic dimensions. The observed decreases in wall thickness and ventricular size in the presence of aortic constriction were probably due to increased phospholamban phosphorylation and improved calcium cycling by I-1c, resulting in de-activation of signaling pathways that contribute to remodeling [41]. These results on long-term improvement of function and remodeling in rodent failing hearts by I-1c are supported by recent studies in a porcine model of heart failure, where I-1c improved contractility and mitigated adverse remodeling [26]. Given the findings in small and large animals, I-1c may represent a molecular inotrope to restore cardiac function and arrest adverse remodeling in failing hearts which exhibit decreased levels and phosphorylation of endogenous inhibitor-1, associated with increased PP1 activity and depressed contractility [14], [15], [40].

In summary, chronic cardiac increases in the expression of I-1c in the pure FVB/N mouse genetic background do not promote cardiomyopathic remodeling or increase susceptibility to catecholaminergic stress upon aging. Additionally, long-term expression of I-1c by gene transfer in failing hearts increases phosphorylation of phospholamban and results in improved function and abrogates adverse remodeling. Taken together, these results support the potential of I-1c as a therapeutic modality in heart failure.
